# Profiling the Phytochemicals of *Orostachys margaritifolia*: Biological Activities, LC-ESI/MS, and HPLC Analyses

**DOI:** 10.3390/ph17030290

**Published:** 2024-02-23

**Authors:** Nari Yoon, Hyeonjun Yu, Gia Han Tran, Chung Ho Ko, Hoon Kim, Mi Jeong Yoon, Sanghyun Lee

**Affiliations:** 1 Department of Plant Science and Technology, Chung-Ang University, Anseong 17546, Republic of Korea; nari990828@naver.com (N.Y.); giahan1121997@gmail.com (G.H.T.); 2 Department of Integrated Biomedical and Life Sciences, Korea University, Seoul 02841, Republic of Korea; ahstmxj77@korea.ac.kr; 3 Division of Garden and Plant Resources, Korea National Arboretum, Pocheon 11186, Republic of Korea; tune0820@korea.kr; 4 Department of Food and Nutrition, Chung-Ang University, Anseong 17546, Republic of Korea; hkim81@cau.ac.kr; 5 Natural Product Institute of Science and Technology, Anseong 17546, Republic of Korea

**Keywords:** ABTS^+^-DPPH radical scavenging activity, HPLC/PDA, LC-ESI/MS, nitric oxide, *Orostachys margaritifolia*

## Abstract

*Orostachys margaritifolia* Y. N. Lee (OMY) is an endemic Korean plant in the family Crassulaceae that is known to contain a variety of bioactive compounds. To assess the physiological activities of an OMY ethanol extract, ABTS^+^ and DPPH radical scavenging assays and a nitric oxide (NO) inhibition assay were conducted. The phytochemical makeup of the extract was profiled via liquid chromatography-mass spectrometry (LC-ESI/MS) and high-performance liquid chromatography with a photodiode array detector (HPLC/PDA). The OMY extract was found to have weaker ABTS^+^ and DPPH radical scavenging activities than the control group (green tea). In the NO inhibition assay, the OMY extract induced a significant increase in macrophage cell viability but showed a lower NO inhibitory activity than l-NAME, producing an IC_50_ value of 202.6 μg/mL. The LC-ESI/MS and HPLC/PDA analyses identified isoquercitrin and astragalin in the OMY extract, quantifying their contents at 3.74 mg/g and 3.19 mg/g, respectively. The study revealed possibilities for the utilization of OMY as a future source of drugs for alleviating inflammation and diseases related to reactive oxygen species.

## 1. Introduction

*Orostachys margaritifolia* Y. N. Lee, of the family Crassulaceae, is an endemic succulent in Jinju, Republic of Korea, where it is known as “Jinjubawisol” [1,2,3]. It has fleshy spatulate leaves, with a spine at the tip, and purplish edges [4]. The Crassulaceae family is morphologically diverse, with more than a thousand species, including succulent plants adapted to barren environments [5,6]. *Orostachys* species are distributed in numerous countries, including China, Korea, and Mongolia [1]. They are also known to have high resistances to adverse weather conditions and pests [7]. The crassulacean acid metabolism (CAM) is a particular mode of photosynthesis in which plants absorb carbon dioxide (CO_2_) at night, increasing water-use efficiency and enabling them to thrive in water-scarce environments [8]. As CAM plants, endemic *Orostachys* species in Korea are highly adapted to their environment, growing and reproducing well in low fertility areas requiring drought and cold tolerance [9]. Additionally, in response to ecological differences caused by environmental conditions, variations such as red dots or borders on leaves are often found within the same species [10].

In recent years, studies on the bioactive compounds found in *Orostachys* species have gained momentum, particularly with a focus on flavonoids. Early investigations, spanning from the early 2000s to recent years, have explored the flavonoid composition of *O. japonicus*, with notable contributions from several researchers [11,12,13]. These efforts have led to the identification of 12 distinct flavonoids in *O. japonicus*. Building upon these findings, Lee et al. [14] expanded the scope, characterizing a total of 16 compounds, including eight flavonoids and one alkaloid, in *O. japonicus*. Additionally, Yin et al. [15] confirmed the presence of various compounds, such as sachaloside A and myrtenyl *O*-β-d-glucoside, in the ethanol (EtOH) extract of *O. malacophyllus* stems. Furthermore, Zhongyi et al. [16] conducted a chemical investigation to identify 28 compounds, including 12 flavonoids, in the EtOH extract of whole plants of on *O. cartilaginea*. Despite these advancements, analytical studies on other *Orostachys* species, such as *O. malacophilus*, remain scarce in the literature. In the present study, LC-electrospray ionization mass spectrometry (LC-ESI/MS) followed by HPLC-diode array detector analysis was used for both the qualitative identification and quantitative determination of key phytochemicals in the *O. margaritifolia* extract.

The most extensively studied pharmacological aspect of the *Orostachys* genus is their anticancer activity, as detailed in recent articles reviewed by Hur et al. [17]. Additionally, scientific evidence suggests that this genus also exhibits various physiological effects, including hepatoprotective [18,19,20], anti-inflammatory [21,22], anti-angiogenic [23], anti-adipogenic [24], anti-diabetic [25], and immunostimulatory [26] activities. However, previous research so far on the pharmaceutical effectiveness of the *Orostachys* genus has primarily focused on one species, *O. japonicus*. Given the remarkable physiological potential demonstrated by the *Orostachys* genus, particularly exemplified by *O. japonicus*, exploring the pharmacological properties of other species, such as *O. margaritifolia*, is essential to broaden the utilization of this genus in plant-derived pharmaceuticals and medicines. Currently, the physiological potential of numerous natural pharmaceuticals for human health is evaluated by examining their antioxidant and anti-inflammatory activities [27,28].

Hence, in this study, radical scavenging analyses and a lipopolysaccharide (LPS)-stimulated nitric oxide determination assay were employed to assess the antioxidant and anti-inflammatory activities, and the data are thought to be considered foundational for potential industrial applications of *O. margaritifolia.*

## 2. Results

### 2.1. LC-ESI/MS

**Figure 1:**
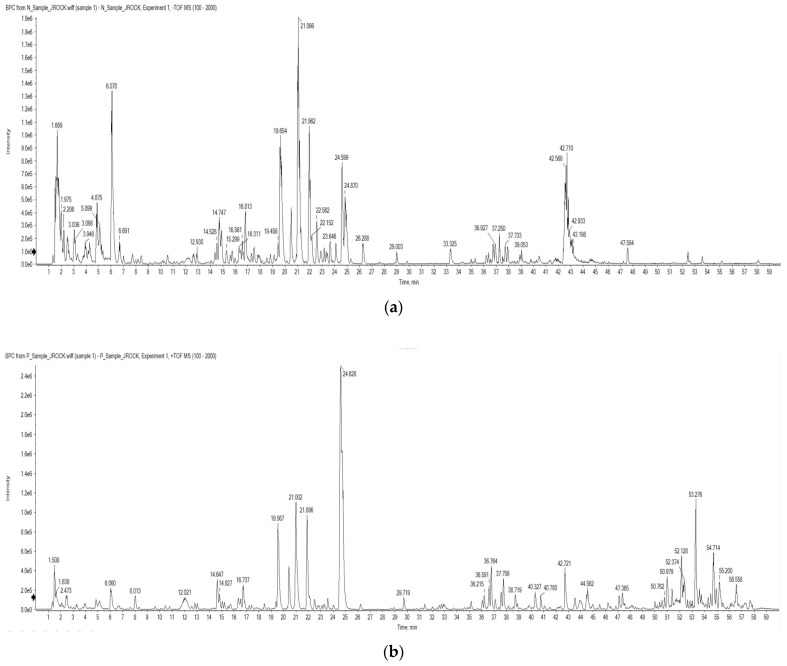
Total ion chromatograms of the OMY extract from an LC-ESI/MS analysis using (**a**) negative and (**b**) positive ionization modes.

**Figure 2:**
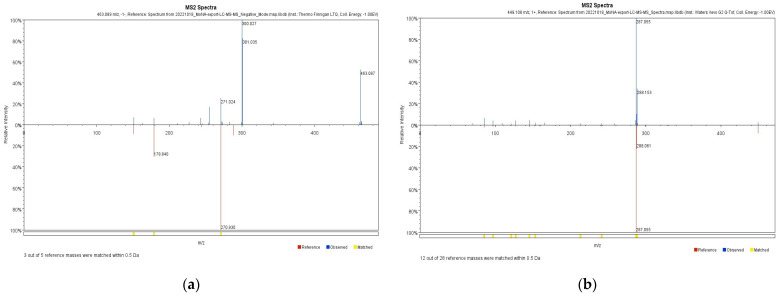
ESI/MS data of (**a**) myricitrin and (**b**) astragalin.

To identify the compound present in OMY, LC-ESI/MS profiling was conducted. The identified compounds are arranged based on their mass-to-charge ratios in Table 1. Myricitrin and astragalin were characterized in both positive and negative ion chromatograms (Figure 1) with retention times of 19.62 and 21.02 min, respectively. The LC-ESI/MS data for myricitrin and astragalin can be seen in Figure 2.

### 2.2. HPLC/PDA Analysis

After LC-ESI/MS profiling, an HPLC/PDA analysis was conducted for further identification and quantification of the compounds. Myricitrin, isoquercitrin and astragalin peaks appeared at the retention time of 10.6, 11.4 and 14.5 min, respectively (Table 2). The calibration curve of the standards showed good linearity, with a correlation factor (*R*-value) of 1. The contents of these phytochemicals in the OMY extract and per g of OMY dry weight (DW) and fresh weight (FW) were calculated (Table 3). Although myricitrin has been characterized in LC-ESI/MS analyses as a major chemical constituent of 

**Figure 3:**
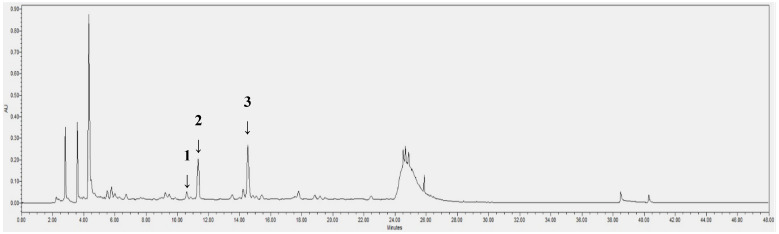
HPLC/PDA chromatogram of the EtOH extract of OMY. The labeled peaks represent myricitrin (**1**), isoquercitrin (**2**) and astragalin (**3**).

OMY, its peak indicated only trace amounts in the HPLC/PDA analysis (Figure 3). However, higher contents of isoquercitrin, 3.74 mg/g and 3.19 mg/g, respectively, were found in the OMY extract. The isoquercitrin content was 1.18 mg/g DW and 0.07 mg/g FW. The content of astragalin was calculated as 1.01 mg/g DW and 0.06 mg/g FW.

### 2.3. Antioxidant Activity

During the ABTS^+^ radical scavenging assay, a concentration range of 3.13–25.00 mg/mL was used for the OMY extract, while lower concentration ranges were used for green tea (0.05–0.39 mg/mL) and AA (0.04–0.20 mg/mL). The IC_50_ values of the OMY extract, green tea, and AA were 10.49 mg/mL, 0.15 mg/mL, and 0.11 mg/mL, respectively (Table 4). Compared to OMY, green tea and AA exhibited stronger antioxidant activity, with similar IC_50_ values. As for the DPPH radical scavenging assay, a concentration range of 1.56–12.50 mg/mL was used for the OMY extract, whereas lower concentration ranges were again used for green tea (0.05–0.39 mg/mL) and AA (0.04–0.20 mg/mL). As a result, IC_50_ values of the OMY extract, green tea, and the AA standard were determined to be 10.49 mg/mL, 0.21 mg/mL, and 0.17 mg/mL, respectively (Table 5). Green tea once again showed a similar IC_50_ value to AA, the standard for DPPH scavenging activity. For both radicals, the antioxidant capacity of AA tended to be strongest, followed by green tea, and then the OMY extract.

### 2.4. NO Inhibitory Activity

**Figure 4:**
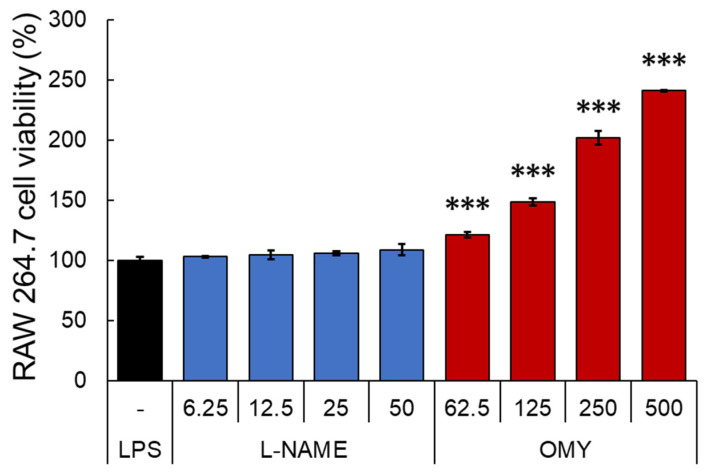
Cytotoxic effects of OMY extract and the drug l-NAME, which inhibits NO production, on lipopolysaccharide (LPS)-stimulated RAW 264.7 cells. The RAW 264.7 cells were treated with samples for 30 min prior to LPS (1 μg/mL) stimulation for 24 h. Cell viability was assessed using conventional MTT assays. Significant differences between the LPS and each other group are represented by asterisks. “***” indicates *p* < 0.001.

**Figure 5:**
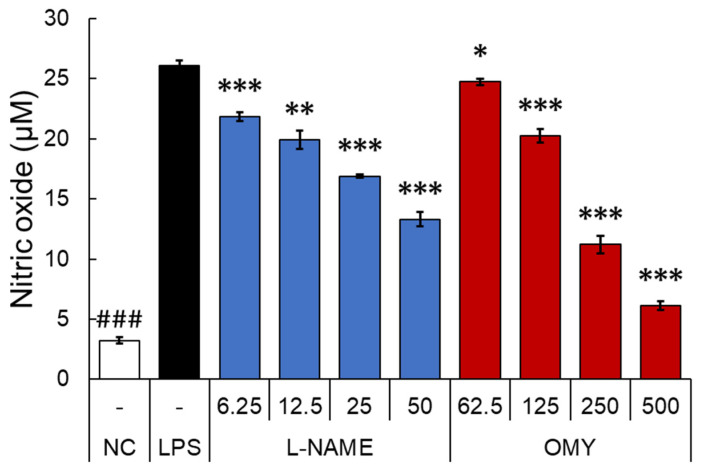
The effect of OMY extract and the drug l-NAME, a non-selective NO synthase inhibitor, on the production of NO in lipopolysaccharide (LPS)-stimulated RAW 264.7 cells. The RAW 264.7 cells were treated with samples for 30 min prior to LPS (1 μg/mL) stimulation for 24 h. The NO concentrations were determined using conventional Griess assays. Different symbols indicate significant differences between the LPS and each other group. (“NC”, denotes the negative control, which received no treatment): *, *p* < 0.05; **, *p* < 0.01; and ### or ***, *p* < 0.001.

After testing the antioxidant activity of OMY extract, the inhibitory activity of NO, a major pro-inflammatory mediator produced by innate immune cells such as macrophage, was evaluated in RAW 264.7 cells stimulated with LPS [29]. Specifically, the cells underwent a 30 min treatment with OMY extract or n-nitro-l-arginine methylester (l-NAME), a non-selective NO synthase inhibitor, followed by LPS stimulation for 24 h [30]. The cytotoxic effects of each sample were then evaluated using MTT assays (Figure 4), and NO production in the culture supernatant was quantified using Griess assays (Figure 5).

Compared to the LPS-treated control group, l-NAME treatment exhibited no impact on cell viability within the concentration range of 6.25–50 μg/mL. Conversely, treatment with the OMY extract at all tested concentrations (62.5–500 μg/mL) demonstrated significant and concentration-dependent increases in viability of 121.5–241.1%, indicating a substantial proliferative effect of the OMY extract on RAW 264.7 cells. Following these findings, we utilized the tested OMY extract concentrations for subsequent experiments. In Figure 4, the effect of the EtOH extract of OMY on excessive NO production induced using LPS-stimulation in RAW 264.7 cells is depicted. In comparison to cells treated with LPS alone (26.1 μM), significant inhibition of NO production was observed in the cells treated with LPS and the OMY extract (24.7–6.1 μM; 5.9–87.4% inhibition) or LPS and l-NAME (21.9–13.3 μM NO, representing 18.5–55.9% inhibition). These findings suggest a roughly fourfold higher NO inhibitory potential for l-NAME compared to the OMY extract (Table 6).

## 3. Discussion

Green tea, which mainly contains catechins, flavonols, tannins and phenolic compounds, is known to have the highest antioxidative activities among different types of tea [31]. However, a study by Kim et al. [32] comparing the ABTS^+^ scavenging activities of three types of tea found that white tea had the strongest activity, followed by green tea and then black tea. Zaiter et al. [33] assessed the correlation between the particle size of green tea leaves and their antioxidant activities and concluded that particle sizes of 100–180 µm, ground at 6000 rpm, had the highest catechin content and the highest radical scavenging capacity. Considering such studies, green tea was used as a control group in the current study. According to an ABTS^+^ radical scavenging assay conducted by Im et al. [34], the ethyl acetate fraction of *O. japonicus* showed weaker antioxidant activity than AA but exhibited radical scavenging activity of more than 50% at a concentration of 0.05 mg/mL. In our data, AA showed the highest scavenging activity with both radicals, and the antioxidant activities of the OMY extract were weaker than those of green tea.

Among the l-arginine analogs that are utilized to hinder NO synthase activity, l-NAME is the most commonly used [35]. Compared to l-NAME, the OMY extract exhibited a lower IC_50_ value (41.1 µg/mL) for NO inhibition. Jeong et al. [21] fractionated a 95% EtOH extract of *O. japonicus* using different organic solvents and found that the dichloromethane fraction had the highest ability to inhibit NO in LPS-stimulated RAW 264.7 cells. Since our experiments were conducted solely with an EtOH extract of OMY, further studies comparing samples prepared in different ways would benefit our understanding of OMY’S chemical composition.

Isoquercitrin (quercetin-3-glucoside) and astragalin (kaempferol-3-glucoside) are two flavonoid compounds that have been isolated from the flower of *Astragalus sinicus* L. [36]. Astragalin has long been utilized for pharmaceutical purposes and is known for its biological functions, such as antioxidant, anti-inflammatory and anticancer activities [37]. Moreover, it is mentionable that astragalin possesses the potential to alleviate osteoporosis, osteoarthritis, and obesity [38]. Among seven compounds found in the shrub *Thuja orientalis*, isoquercitrin was notable for its strong antioxidant activity [39,40,41,42,43]. An in vitro test of isoquercitrin and an in vivo test using cadmium-treated mouse kidney and liver cells, conducted by Li et al. [44] indicated that isoquercitrin has the potential to resist cadmium toxicity. Myricitrin, isolated as a major compound from *Myrcia splendens* and *M. palustris*, has been studied for its strong ability to ameliorate toxic liver damage [45,46]. Myricitrin also showed mentionable antithrombotic effects in vitro and in a rat acute blood stasis model in vivo [47]. Ma et al. [11] isolated and identified seven compounds from the *n*-butanol fraction of *O. japonicus*, including astragalin and isoquercitrin. According to an HPLC/PDA analysis conducted by Nugroho et al. [2], isoquercitrin was identified as one of the main peaks produced from an OMY MeOH extract, with a content of 5.12 mg/g, whereas our EtOH extract had a slightly lower content of 3.74 mg/g included in the EtOH extract.

## 4. Materials and Methods

### 4.1. Plant Materials

**Figure 6:**
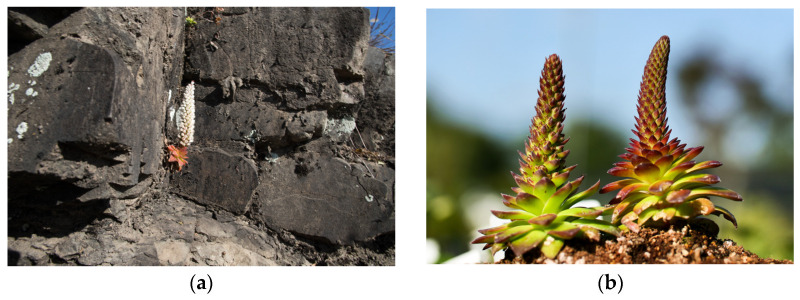
Natural habitat (**a**) and experimental samples (**b**) of OMY.

*Orostachys margaritifolia* Y. N. Lee (OMY) seeds were collected from an adult individual around Jinyangho Lake in Panmundong, Jinju, Korea in August 2021. After collection, the plants were grown in a greenhouse at the Korea National Arboretum’s Plant Resources Conservation Center, Pocheon, Korea. For two years, until August 2023, no fertilizer was supplied, only water, and the light conditions were optimal. No pests occurred during cultivation (Figure 6).

The plant materials used in the experiment were in the adult stage with all the main leaves fully developed. A voucher specimen (No. LEE23-05) was deposited at the herbarium of the Department of Plant Science and Technology, Chung-Ang University, Anseong, Republic of Korea.

### 4.2. Instruments and Reagents

**Figure 7:**
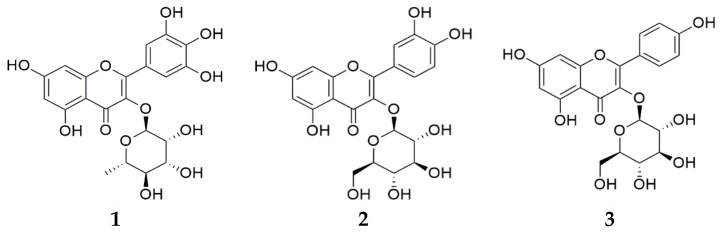
Chemical structures of (**1**) myricitrin, (**2**) isoquercitrin, and (**3**) astragalin.

The LC-ESI/MS analysis was conducted using a Thermo Vanquish UHPLC (Thermo Scientific, San Jose, CA, USA) and a high-resolution mass spectrometer (Q Exactive Hybrid Quadrupole-Orbitrap, Thermo Scientific, San Jose, CA, USA). The quantitative analysis was conducted using an HPLC system (Waters Alliance e2695 Separations Module, Miliford, MA, USA) consisting of an auto-sampler, pump, and photodiode array detector (Waters 2998 PDA detector, Milford, MA, USA). The HPLC-grade solvents included water and acetonitrile (ACN) purchased from J. T. Baker (Phillipsburg, PA, USA), and HPLC-grade trifluoroacetic acid (TFA) purchased from Thermo Scientific (Waltham, MA, USA). Myricitrin, isoquercitrin, and astragalin (Figure 7) were obtained from the Natural Product Institute of Science and Technology (www.nist.re.kr: accessed on 4 Decmber 2023), Anseong, Republic of Korea.

### 4.3. Extraction from OMY Samples

A fresh OMY sample (70.5 g) was placed in a deep freezer for lyophilization, resulting in a dried sample (4.4 g). The dried OMY sample was ground into a fine powder and extracted using a Soxhlet reflux evaporator with 132 mL of 95% EtOH. Each extraction was carried out for 3 h, and repeated thrice, followed by evaporation using a rotary evaporator, resulting in the collection of an EtOH extract (1.4 g).

### 4.4. Preparation of Samples and Standard Solutions for HPLC/PDA

In total, 30 mg of the EtOH extract of OMY was dissolved in 1 mL of methanol (MeOH) to create a sample stock solution. As standards, myricitrin, isoquercitrin, and astragalin were also individually dissolved in MeOH (1 mg/mL). Isoquercitrin and astragalin were sequentially diluted to 500, 250, 125, 62.5, 31.25 and 15.63 ppm for quantitative analysis. Each sample and standard were sonicated before use.

### 4.5. LC-ESI/MS Conditions

Chromatographic separation was executed using an LC system comprising a Thermo Vanquish UHPLC equipped with a Waters Cortex T3 column (150 mm × 2.1 mm, particle size 1.6 μm), maintained at a temperature of 45 °C. The flow rate was fixed at 0.25 mL/min. The mobile phase consisted of 0.1% HCOOH in water (A) and 0.1% HCOOH in ACN (B). Employing a gradient mode, the initial composition started at 3% B, increased to 15% B over 15 min, further elevated to 100% B over the subsequent 35 min, and held for an additional 5 min, resulting in a total run time of 55 min. Subsequently, the column was re-equilibrated with a 3% B solution for 5 min. The MS analysis was conducted utilizing a high-resolution mass spectrometer equipped with a heated electrospray ion source (H-ESI) operated in both positive and negative ion modes. A full-scan MS spectrum (*m*/*z* 100–1500) was acquired using a quadrupole system with a resolution setting of 70,000. The spray voltage was adjusted to 3.5 kV for the cation mode and 3.0 kV for the anion mode. The top 10 most intense precursor ions were selected for MS2 fragmentation, and spectrum acquisition was carried out with a resolution setting of 17,500. Other relevant MS parameters included a capillary temperature of 320 °C, sheath gas flow rate of 50 AU, sweep gas flow rate of 1 AU, and auxiliary gas flow rate of 10 AU.

### 4.6. HPLC/PDA Conditions

An HPLC/PDA analysis of the OMY extract was performed using a YMC Pack Pro C18 column (4.6 × 250 mm, 5 µm) as the stationary phase. The mobile phase consisted of 0.1% of phosphoric acid in water (A) and ACN (B). The flow rate was maintained at 1 mL/min, the column oven temperature was set to 35 °C, the injection volume was 10 µL, and the detector wavelength was 265 nm. The analysis was conducted using a linear gradient elution protocol: 17% B at 0 min, increasing to 27% B at 20 min, then increasing to 90% B at 25 min, held at 90% B until 35 min, decreasing back to 17% B at 36 min, and held at 17% B until 48 min.

### 4.7. Calibration Curve

In forming the calibration curve, the value of the X (μg/mL) signifies the concentration of the standard, and the Y axis value (mAU) indicates the area of the standards (Table 2). The total isoquercitrin and astragalin contents (mg/g) were calculated by multiplying C, V, D, P and dividing by W (C: concentration of standard, V: total volume of the test solution, D: dilution factor, P: standard purity, W: sample weight).

### 4.8. ABTS^+^ Radical Scavenging Activity

To assess the 2,2′-azinobis-(3-ethylbenzothiazoline-6-sulfonic acid (ABTS^+^) radical-scavenging activity of OMY, 50 mg of the OMY extract was dissolved in water to create a test solution. A total of 10 µL of the OMY test solution were then added to wells in the 96-well plate, followed by the addition of 200 µL of ABTS^+^ working solution. After mixing with a microplate shaker and incubating for 30 min in a dark room, the absorbance was measured at 734 nm using a microplate reader. Each application was repeated thrice and the ABTS^+^ working solution was replaced with water for a blank test. Ascorbic acid (AA) was used as the standard and green tea extract was used as the control group.

### 4.9. DPPH Radical Scavenging Activity

A total of 50 mg of the OMY extract was dissolved in EtOH and filtered using a polyvinylidene fluoride filter to create a test solution. An amount of 10 mg of the test solution and 200 µL of DPPH working solution were then added to wells in a 96-well plate. After mixing and a 30 min dark incubation, the absorbance was measured at 514 nm using a microplate reader. This progress was repeated three times. For a blank test, the DPPH working solution was replaced with 95% EtOH, AA and green tea extract were used as the standard and control group, respectively.

### 4.10. Nitric Oxide (NO) Inhibitory Activity

The murine macrophage cell line RAW 264.7, obtained from the Korean Cell Line Bank (KCLB) in Seoul, South Korea, was maintained in Dulbecco’s modified Eagle medium (DMEM) supplemented with 10% fetal bovine serum (FBS) and 1% penicillin-streptomycin. Cultures were incubated at 37 °C in a 5% CO_2_ atmosphere and subcultured every 2–3 days. Aliquots (200 μL) of cell suspension at densities of 3 × 10^5^ cells/well were incubated in a 96-well culture plate (Corning) until reaching 80–85% confluence on the bottom of plate. After removing the culture supernatant, serum-free DMEM containing 1% penicillin-streptomycin (without FBS) and the OMY extract sample was added and incubated for 30 min. Afterward, lipopolysaccharide (LPS; *Escherichia coli* origin; Sigma-Aldrich, St. Louis, MO, USA) was added to stimulate NO production. Following incubation for 24 h, the cytotoxic effect was evaluated using the conventional 3-(4,5-dimethylthiazol-2-yl)-2,5-diphenyl-tetrazolium bromide (MTT) assay as previously described [48]. Results were presented as the percentage viability relative to the LPS-treated control group. NO levels in the cell culture supernatant were assessed using the Griess assay with a commercial kit (Thermo Fisher Scientific), and the results were expressed as the half maximal inhibitory concentration (IC_50_) value.

### 4.11. Statistical Analysis

Statistical analyses involved Student’s *t*-tests or one-way ANOVAs, followed by Tukey’s post hoc tests. All analyses were conducted using Predictive Analytics Software (PASW^®^; v12.0) Statistics 18 (IBM Co., Armonk, NY, USA). The outcomes are presented as means ± standard deviation (SD), and *p*-values < 0.05 were considered significant in all tests.

## 5. Conclusions

This study focused on identifying marker compounds in OMY to assess its potential for future utilization in the pharmaceutical industry, particularly for alleviating inflammation and scavenging free radicals. For this reason, ABTS^+^ and DPPH radical scavenging assays and an NO inhibition assay were conducted, and LC-ESI/MS and HPLC/PDA analyses were performed to identify and quantify the major components of an EtOH extraction of OMY. The results showed that OMY extract exhibited weaker antioxidant capacity than green tea, a well-known source of strong antioxidant activity. For instance, the IC_50_ values of green tea for the ABTS^+^ and DPPH radical scavenging assays were 0.15 mg/mL and 0.21 mg/mL, respectively, whereas the IC_50_ values of the OMY extract appeared as 10.49 mg/mL and 10.31 mg/mL, respectively. In anti-inflammatory experiments, it exhibited a significant concentration-dependent increase in the viability of RAW 264.7 macrophage cells, but a lower inhibitory ability against NO compared to l-NAME. For l-NAME, the IC_50_ value for NO inhibition in LPS-stimulated RAW 264.7 cells was 41.1 μg/mL, while OMY extract produced an IC_50_ value of 202.6 μg/mL. The LC-ESI/MS and HPLC/PDA analyses revealed the presence of myricitrin, isoquercitrin, and astragalin. The content of isoquercitrin and astragalin was calculated through quantitative analysis, measuring 3.74 mg/g and 3.19 mg/g, respectively. However, further experiments on the bioactive properties of OMY such as anti-cancer and anti-diabetes activities, and toxicity tests using in vivo assays, are needed to further substantiate its potential for use in the pharmaceutical industry.

## Figures and Tables

**Table 1:** Compounds identified via LC-ESI/MS profiling of the OMY extract using both positive and negative ionization modes.

Retention Time (min)	Molecular Weight	Proposed Compound
2.51	332.1	Galloyl glucose ^2^
5.23	332.1	Gallic acid hexoside ^2^
12.05	449.1	Cyanidijin-3-*O*-glucoside ^1^
12.60	356.1	1-*O*-Feruloylglucose ^2^
12.88	626.1	Quercetin-3,4′-diglucoside ^1,2^
14.69	610.2	Luteolin-3′,7-di-*O*-glucoside ^1,2^
14.90	610.2	Luteolin-3′,7-di-*O*-glucoside ^2^
16.19	626.1	Herbacetin-3,8-diglucoside ^2^
16.50	596.1	Peltatoside ^1^
16.56	596.1	Rhodalidin ^2^
16.80	594.2	Kaempferol-3-neohesperidoside ^1^
17.11	464.1	Myrtillin ^2^
17.26	596.1	Peltatoside ^1,2^
19.62	464.1	Myricitrin ^1,2^
19.82	610.2	Luteolin-3′,7-di-*O*-glucoside ^2^
20.48	550.1	Quercetin-3-*O*-malonylglucoside ^2^
20.91	506.1	Quercetin-3-(6″-acetylglucoside) ^2^
21.02	448.1	Astragalin ^1,2^
21.12	448.1	Kaempferol-7-*O*-glucoside ^2^
21.94	534.1	Luteolin-7-(6″-malonylglucoside) ^1,2^
22.52	432.1	Afzelin ^1,2^
24.11	302.0	Quercetin ^2^
26.29	286.0	Kaempferol ^2^

^1^ positive ion mode. ^2^ negative ion mode.

**Table 2:** Calibration curves for myrictrin (**1**), isoquercitrin (**2**), and astragalin (**3**) of OMY.

Compound	t_R_ ^a^	Calibration Equation ^b^	*R*-Value ^c^
**1**	10.6	-	-
**2**	11.4	Y = 26,898X − 13,035	1
**3**	14.5	Y = 21,602X − 25,971	1

^a^ retention time. ^b^ Y = peak area, X = concentration of the standard (µg/mL). ^c^ *R*-value = correlation coefficient for five data points in the calibration curve.

**Table 3:** Contents of myricitrin (**1**), isoquercitrin (**2**), and astragalin (**3**) in the OMY extract and plant tissues, expressed per g of dry weight (DW) and fresh weight (FW).

Compound	mg/g Extract	mg/g DW	mg/g FW
**1**	tr ^a^	tr	tr
**2**	3.74 ± 0.01	1.18 ± 0.00	0.07 ± 0.00
**3**	3.19 ± 0.02	1.01 ± 0.01	0.06 ± 0.00

^a^ trace amount.

**Table 4:** The ABTS^+^ radical scavenging activity of green tea, OMY extract, and an ascorbic acid (AA) standard.

Sample	Concentration (mg/mL)	Scavenging Activity (%)	IC_50_ (mg/mL)
Green tea	0.05	19.32 ± 3.90	0.15 ± 0.01
	0.10	36.57 ± 4.27	
	0.20	62.84 ± 2.68	
	0.39	92.86 ± 0.46	
OMY	3.13	25.65 ± 3.39	10.49 ± 1.32
	6.25	40.75 ± 3.13	
	12.50	55.22 ± 1.91	
	25.00	78.20 ± 2.37	
AA	0.04	15.80 ± 4.07	0.11 ± 0.01
	0.08	34.60 ± 1.57	
	0.12	56.44 ± 3.81	
	0.16	75.57 ± 4.33	
	0.20	93.75 ± 1.53	

**Table 5:** The DPPH radical scavenging activity of green tea, OMY extract, and an ascorbic acid (AA) standard.

Sample	Concentration (mg/mL)	Scavenging Activity (%)	IC_50_ (mg/mL)
Green tea	0.05	10.00 ± 0.90	0.21 ± 0.00
	0.10	25.30 ± 1.81	
	0.20	51.71 ± 1.07	
	0.39	84.74 ± 0.89	
OMY	1.56	16.83 ± 3.00	10.31 ± 0.35
	3.13	24.56 ± 0.82	
	6.25	36.17 ± 1.11	
	12.50	57.59 ± 1.59	
AA	0.04	4.63 ± 1.67	0.17 ± 0.00
	0.08	17.22 ± 0.41	
	0.12	34.37 ± 0.53	
	0.16	46.41 ± 3.18	
	0.20	63.09 ± 0.94	

**Table 6:** The IC_50_ values for nitric oxide (NO) inhibition in LPS-stimulated RAW 264.7 cells treated with OMY extract or the drug l-NAME.

Sample	NO Inhibition (IC_50_, μg/mL)
l-NAME	41.1 ± 2.6
OMY	202.6 ± 8.7

## Data Availability

Data is contained within the article.
